# Malignant Intermittent Cured without Bark

**Published:** 1829-07-01

**Authors:** 


LVII.

### PHILADELPHIA INFIRMARY.

MALIGNANT INTERMITTENT. By Dr. JACKSON.

The following case, reported in the American Journal of the Medical Sciences, is worthy of notice, as shewing that very severe malarious intermittents will give way to low diet, change of air, and moderate local depletion, even without bark.

"*Case*. Chas. Cavenaugh, ætat. twenty-two, was admitted about 12 o'clock, reporting that he had intermittent fever. He had been engaged during the Summer at work on the canal at the Juniata, where he had contracted the disease; and of a gang of three hundred who left the city, he stated more than half were dead. It was originally tertian: for the last week it had assumed the quotidian type, coming on at two o'clock. This change has been effected by fatigue in travelling from the Juniata. Habits intemperate.

"When the chill was subsiding about two o'clock to-day, the fever commenced with the sensation of a violent blow upon the forehead, and immediately he jumped from his bed, and ran about the ward with the wildness of maniacal delirium. Being overtaken and replaced, he appeared ignorant, on becoming better, that he had left his bed. At this period he was found labouring under a most violent excitement; he appeared almost distracted with the pain in his head; his respiration was exceedingly laborious; the skin very hot; and the pulse full, strong, and frequent; ℥xij. of blood were immediately taken from his arm, and in a few minutes after he was completely relieved. 7 p. m. asleep, with his head covered; woke up alarmed; skin very hot; pulse full and frequent.

"5th.—Better this morning; the intermission is not complete; skin hot; pulse frequent; tongue moist and furred; eyes injected and jaundiced; absolute diet; hot revulsives to anticipate chill; the paroxysm in the afternoon was nearly as violent as that of yesterday; required two men to hold him in bed; his spleen was so engorged that it rose in a large ball under the hand; having some tenderness at the epigastrium, and a dry, furred tongue, he was ordered to be cupped freely.

"6th.—A tolerable intermission early this morning; tongue nearly clean. Ordered to take in solution a grain of sulphate of quinine every hour. Paroxysm came on an hour and a half earlier to-day; not so violent; mustard plasters were applied to his extremities when the chill threatened; tongue very much furred in the evening. Ordered cups again to epigastrium.

"7th.—His stomach still irritable; tongue foul; omit quinine; sinapisms when chill threatens. 9 p. m. Had no paroxysm this day.

"Discharged well, having had no return.—Case reported by Dr. JONES.

"*Remarks*.—This case was an intermittent of malignant character, threatening apoplexy in the paroxysm. The intemperate habits of this man, it is most probable, gave to his brain the predisposition which invested its sympathetic irritation when, in the febrile stage, it began to be irradiated from the stomach, with its extreme intensity.

"A full bleeding immediately arrested the violence of the cerebral irritation, and diminished the congestion of the brain. The same remedy would have been more freely urged, but for the habits of the patient, which always counter-indicate the very liberal employment of the lancet. The intemperate are very soon prostrated by general bleeding, and when they once begin to sink, it is almost impossible to re-excite them. This is one of the causes that render inflammations, and diseases requiring free depletion in their treatment, so generally fatal in those who abuse the employment of ardent spirits. They will not bear the treatment which can alone cure them.

"A circumstance in this case to be remarked, is the extraordinary suddenness with which the cerebral irritation commenced, and consequent raptus of blood to the brain. Cavenaugh insisted on it, he had received a blow, and for a time was unconscious of his situation. We have here presented the mode in which apoplexies are induced, and which so commonly draw the cerebral irritation on which they depend, from gastric irritation.

"It is also to be observed that the sulphate of quinine was not tolerated by the stomach, the irritation of which it augmented, and that the cure was effected chiefly by attacking the gastric irritation, by low diet, cooling demulcent drinks, and local depletion."

---

